# Response of the organellar and nuclear (post)transcriptomes of Arabidopsis to drought

**DOI:** 10.3389/fpls.2023.1220928

**Published:** 2023-07-17

**Authors:** Duorong Xu, Qian Tang, Ping Xu, Anton R. Schäffner, Dario Leister, Tatjana Kleine

**Affiliations:** ^1^ Plant Molecular Biology, Faculty of Biology, Ludwig-Maximilians-University Munich, Planegg-Martinsried, Germany; ^2^ Department of Environmental Sciences, Institute of Biochemical Plant Pathology, Helmholtz Zentrum München, München, Germany

**Keywords:** alternative splicing, carbonic anhydrase, chloroplast, drought, *FLOWERING LOCUS M* (FLM), mitochondria, nucleus, (post)transcriptome

## Abstract

Plants have evolved sophisticated mechanisms to cope with drought, which involve massive changes in nuclear gene expression. However, little is known about the roles of post-transcriptional processing of nuclear or organellar transcripts and how meaningful these changes are. To address these issues, we used RNA-sequencing after ribosomal RNA depletion to monitor (post)transcriptional changes during different times of drought exposure in Arabidopsis Col-0. Concerning the changes detected in the organellar transcriptomes, chloroplast transcript levels were globally reduced, editing efficiency dropped, but splicing was not affected. Mitochondrial transcripts were slightly elevated, while editing and splicing were unchanged. Conversely, alternative splicing (AS) affected nearly 1,500 genes (9% of expressed nuclear genes). Of these, 42% were regulated solely at the level of AS, representing transcripts that would have gone unnoticed in a microarray-based approach. Moreover, we identified 927 isoform switching events. We provide a table of the most interesting candidates, and as proof of principle, increased drought tolerance of the carbonic anhydrase *ca1* and *ca2* mutants is shown. In addition, altering the relative contributions of the spliced isoforms could increase drought resistance. For example, our data suggest that the accumulation of a nonfunctional *FLM* (*FLOWERING LOCUS M*) isoform and not the ratio of *FLM-ß* and *-δ* isoforms may be responsible for the phenotype of early flowering under long-day drought conditions. In sum, our data show that AS enhances proteome diversity to counteract drought stress and represent a valuable resource that will facilitate the development of new strategies to improve plant performance under drought.

## 1 Introduction

Land plants must cope with all sorts of environmental conditions, since, as sessile organisms, they cannot evade them. Owing to climate change, the frequency and amplitude of extreme conditions are increasing, and this seriously threatens crop yields worldwide (Zhao et al., 2017). Drought is the most important abiotic stress (Singh et al., 2018), and responses to drought are influenced by developmental stage, plant species and degree of stress (Hu and Xiong, 2014). The primary strain during drought stress is dehydration – loss of water from the cell – which triggers osmotic and hormone-related signals (Blum, 2016), in particular the phytohormone abscisic acid (ABA) (Zhu, 2002). Terminology and approaches to drought research vary widely and the issues are often oversimplified (Lawlor, 2013; Blum and Tuberosa, 2018; Kooyers, 2019). Taking our cue from the work of Lawlor (2013) and Blum (2016) and the TNAU Agritech Portal (https://agritech.tnau.ac.in/agriculture/agri_drought_tolerent_mechanism.html), we define drought resistance in terms of the following features: (i) drought survival, ii) drought escape, iii) drought avoidance, and iv) drought tolerance. Drought survival refers to the fact that, under drought conditions, cells, tissues, and organs are able to maintain key cellular functions and can recover, with minimal damage, upon relief of drought stress (Lawlor, 2013). Dehydration avoidance is a response to moderate, temporary drought stress, which involves deposition of cuticular waxes and growth retardation, in addition to the reduction of transpiration *via* ABA-mediated closure of stomata (Gupta et al., 2020). In the drought-escape strategy, which is employed by annual plants like *Arabidopsis thaliana* (Arabidopsis), flowering is accelerated before drought can compromise survival of the plant (Kenney et al., 2014). In drought tolerance, the plant is able to maintain its functions under dehydration – for example, by producing larger amounts of sugars, osmoprotectants, antioxidants, and scavengers of reactive oxygen species (Hu and Xiong, 2014).

Responses to drought and other stresses are accompanied by large-scale changes in gene expression that facilitate the production of compounds needed to counteract the imposed strain (Bashir et al., 2019). Thus, transcriptome-based studies have identified acetate as a compound that helps plants to survive drought stress (Kim et al., 2017), and shown that the transcription factor AGAMOUS-LIKE 22 (also known as SHORT VEGETATIVE PHASE, SVP), which participates in primary metabolism and developmental processes, is activated in drought-stressed Arabidopsis plants (Bechtold et al., 2016). Here it has to be noted that these experiments were performed under short-day conditions.

Changes in nuclear gene expression in response to environmental perturbations, including drought stress, are fine-tuned by retrograde signals from the chloroplast (Estavillo et al., 2011; Kleine and Leister, 2016), and chloroplasts act both as a target and as a sensor of environmental changes (Kleine et al., 2021). In a forward genetic screen two chloroplast proteins involved in drought resistance were identified (Hong et al., 2020). Furthermore, the first steps of ABA biosynthesis take place in plastids (Ali et al., 2020), which underlines the importance of the chloroplast in the response to drought stress. Although chloroplasts and mitochondria each harbor only around 100 genes (Kleine et al., 2009), organellar gene expression is complex, and transcripts can undergo post-transcriptional modification events such as splicing and editing (Germain et al., 2013). Therefore, the abundance and functionality of a number of organellar proteins depends not only on the levels of their transcripts, but also on their modification status. The same holds true for nucleus-encoded transcripts: Here, alternative splicing (AS) provides for the synthesis of different transcript isoforms from the same gene, thereby increasing proteome diversity (Laloum et al., 2018; Bashir et al., 2019). It has become clear that AS is of central importance for abiotic stress tolerance in plants (Laloum et al., 2018), and AS itself is dynamically regulated under cold stress (Calixto et al., 2018). Concerning organellar transcriptome changes, it has been demonstrated that heat stress over periods of several hours increases the abundance of chloroplast transcripts, and induces a global reduction in splicing and editing efficiency (Castandet et al., 2016), but to our knowledge, the behavior of the mitochondrial (post)transcriptomic landscape has not been investigated so far.

However, microarray analysis has been the major source of information on transcriptome changes in response to drought (Bechtold et al., 2016; Kim et al., 2017; Bashir et al., 2019), while transcriptome-wide AS analysis in response to cold has been addressed with the aid of mRNA sequencing (mRNA-Seq) (Calixto et al., 2018). Both methods make use of samples enriched for polyadenylated transcripts, and are designed specifically to trace the accumulation of nuclear transcripts. Functional organellar transcripts are not polyadenylated; indeed, unlike nuclear transcripts, they become unstable upon addition of a poly(A) tail (Rorbach et al., 2014).

Therefore, we set out to extend the investigation of changes in nuclear gene expression under drought stress by undertaking an overarching characterization of the post(transcriptome), including alternative splicing of nuclear transcripts, accumulation of organellar (chloroplast and mitochondrion) transcripts, and editing and splicing of organellar transcripts. To this end, an RNA-Seq strategy was applied, which involves library preparation after depletion of ribosomal RNAs instead of enrichment for mRNAs as in mRNA-Seq. For reproducibility, an already published drought kinetics set-up (Kim et al., 2017) was applied. Based on our platform, we describe changes in the various organellar and nuclear (post)transcriptomes under drought and complement this with an analysis of nuclear-encoded transcript isoform switches. We provide a table with the most interesting candidates worth for the investigation of their involvement in drought responses. In particular, we demonstrate increased drought tolerance of the carbonic anhydrase *ca1* and *ca2* mutants, and suggest an FLM (FLOWERING LOCUS M)-dependent early flowering mechanism under long-day drought conditions, which is characterized by massive production of non-functional *FLM* isoforms.

## 2 Materials and methods

### 2.1 Plant material and growth conditions

All Arabidopsis (*Arabidopsis thaliana*) lines used in this study share the Columbia genetic background. The carbonic anhydrase *ca1* and *ca2* mutants were described previously (Dabrowska-Bronk et al., 2016). Seeds were sown on 1/2 MS medium containing 1% (w/v) sucrose and 0.8% (w/v) agar, incubated at 4°C for 2 d, and transferred to a climate chamber under a 16-h-light/8-h-dark cycle with a light intensity of 100 μE m^-2^ s^-1^ at 22°C. For physiological experiments, the plants were grown on potting soil (A210, Stender, Schermbeck, Germany) for 3 or 4 weeks.

For drought treatments, 7-day-old seedlings were transferred to pots, and grown for a further 2 weeks under normal growth conditions. Three-week-old plants were subjected to drought conditions by withholding water for the indicated times. The drought phenotypes were documented with a camera (Canon 550D, Krefeld, Germany). For strictly controlled drought experiments, seeds were stratified for 3 days, and then sown directly on soil for germination. After 5 days, seedlings were transferred to pots containing 100 g moist soil and sand mixture (150 ± 0.5 g total pot weight) with 5 replicates for each genotype in a randomized design. From this time on, plant growth was in controlled environment on a conveyor-belt organized system allowing programmable watering by pot weight, RGB imaging, and chlorophyll fluorescence analyses (Photon Systems Instruments, Ltd. (PSI), Drasov, Czech Republic); there, plants were grown under short-day conditions (10 h light, 22°C, 45% relative humidity/14h darkness, 20°C, 60% relative humidity; Georgii et al., 2017) with LED (white, blue, red, dark red) illumination at a light intensity of 115 μE m^−2^ s^−1^. The pot weight was increased to 165 g within two days by multiple watering (3 g a time, 2-3 times a day). Drought treatment began for 18-day-old plants. During the drought treatment, the pots lost approximately 3 g/day until pot weight reached 110 g (age of the plants: 39 days). Pots were weighed daily and watered automatically to ensure consistent weight loss for each pot. Drought treatment was terminated when the Fv/Fm value for Col-0 plants were close to zero (44-day-old plants, when pot weight was approximately 110 g). The pots were then gradually re-watered to 130 g over a week for recovery, then survival rates were calculated for each genotype. Throughout the growth period in the PSI system, all parameters were automatically recorded. Surviving plants were monitored by determination of the Fv/Fm value.

### 2.2 Nucleic acid extraction

Leaf tissue (100 mg) was homogenized in extraction buffer containing 200 mM Tris/HCl (pH 7.5), 25 mM NaCl, 25 mM EDTA and 0.5% (w/v) SDS. After centrifugation, DNA was precipitated from the supernatant by adding isopropyl alcohol. After washing with 70% (v/v) ethanol, the DNA was dissolved in distilled water.

For RNA isolation, plant material was harvested, frozen in liquid nitrogen and crushed in a TissueLyser (Retsch, model: MM400). Total RNA was extracted with the Direct-zol RNA Kit (Zymo Research, Irvine, USA) according to the manufacturer’s protocol. RNA samples were quantified with a Nanodrop spectrophotometer (ThermoFisher Scientific, Langenselbold, Germany) and RNA integrity and quality were assessed with an Agilent 2100 Bioanalyzer (Agilent, Santa Clara, USA). Isolated RNA was stored at −80°C until further use.

### 2.3 cDNA synthesis and quantitative reverse transcriptase-polymerase chain reaction analysis

One microgram of RNA was employed to synthesize cDNA using the iScript cDNA Synthesis Kit (Bio-Rad, Munich, Germany). Quantitative real-time PCR analysis was performed on a CFX Connect™ Real-Time PCR Detection System (Bio-Rad) with the SsoAdvanced™ Universal SYBR^®^ Green Supermix (Bio-Rad). The gene-specific primers used for this assay are listed in **Supplementary Table 1** in the **Supplementary Material file**. Each sample was quantified in triplicate and normalized using *AT3G58500* (which codes for protein phosphatase 2A-4 and showed minimal expression changes during drought treatment) as an internal control.

### 2.4 RNA-sequencing

Total RNA from plants was isolated using TRIzol Reagent™ (Thermo Fisher Scientific, Waltham, MA, USA) and purified using an RNA Clean & Concentrator (Zymo Research, Irvine, USA) according to the manufacturer’s instructions. RNA integrity and quality were assessed with an Agilent 2100 Bioanalyzer (Santa Clara, USA). RNA was depleted of ribosomal RNA with the RiboMinus Plant Kit for RNA-seq (Thermo Fisher Scientific), and rRNA-free RNA was cleaned by ethanol precipitation. The directional library was prepared at Novogene Biotech (Beijing, China) with the help of the NGS Stranded RNA Library Prep Set (Novogene Biotech, PT044): The purified RNAs were first fragmented randomly to short fragments of 150-200 bp by addition of fragmentation buffer, followed by cDNA synthesis using random hexamers. After the first strand was synthesized, second-strand synthesis buffer (Illumina), dNTPs (dUTP, dATP, dGTP and dCTP) and DNA polymerase I were added to synthesize the second-strand. This was followed by purification by AMPure XP beads, terminal repair, polyadenylation, sequencing adapter ligation, size selection and degradation of second-strand U-contained cDNA by the USER enzyme. The libraries were checked with Qubit and real-time PCR for quantification and an Agilent 2100 Bioanalyzer (Santa Clara, USA) for size distribution detection. 150-bp paired-end sequencing was conducted on an Illumina HiSeq 2500 system (Illumina, San Diego, USA) at Novogene Biotech with standard Illumina protocols. Three independent biological replicates were used per time point (0, 6, 9, 12 and 15 days). However, sequencing data from one sample (Col-0 subjected to 6 days of drought treatment) were not satisfactory. In this case, two replicates were used. Sequencing data have been deposited to Gene Expression Omnibus (Edgar et al., 2002) under the accession number GSE202931.

### 2.5 Chloro-Seq analysis

To detect instances of editing and splicing of organellar transcripts in our RNA-Seq data, a modified Chloro-Seq pipeline (Malbert et al., 2018) was used. The RNA-Seq reads were processed on the Galaxy platform (https://usegalaxy.org/) to remove the adaptors by Trimmomatic (Bolger et al., 2014), then sequencing quality was assessed with FastQC (http://www.bioinformatics.babraham.ac.uk/projects/fastqc/). The trimmed reads were mapped using RNA STAR (Dobin et al., 2013) with TAIR10_Chr.all.fasta (https://www.arabidopsis.org/download_files/Genes/TAIR10_genome_release/TAIR10_chromosome_files/TAIR10_chr_all.fas) as reference genome file, and Araport11_GFF3_genes_transposons.201606.gff (https://www.arabidopsis.org/download_files/Genes/Araport11_genome_release/archived/Araport11_GFF3_genes_transposons.201606.gff.gz) as gene model file for splice junctions. The mapped bam file was later used as the input for Chloro-Seq analysis (https://github.com/BenoitCastandet/chloroseq) to determine editing and splicing efficiency.

### 2.6 3D RNA-Seq analysis

To quantify overall transcript accumulation and detect alternative splicing (AS) of nuclear transcripts, the 3D RNA-Seq pipeline (Guo et al., 2021) was used. To this end, RNA-Seq reads were prepared on the Galaxy platform (https://usegalaxy.org/). Adaptors were removed with Trimmomatic (Bolger et al., 2014), and sequencing quality was accessed with FastQC (http://www.bioinformatics.babraham.ac.uk/projects/fastqc/). Transcript abundances were calculated using Salmon (Patro et al., 2017) and AtRTD2-QUASI as reference transcriptome (Zhang et al., 2017). The generated files were uploaded into the 3D RNA-Seq app (https://3drnaseq.hutton.ac.uk/app_direct/3DRNAseq; Calixto et al., 2018; Guo et al., 2021) and transcript per million reads (TPMs) were calculated using the implemented lengthScaledTPM method. Weakly expressed transcripts and genes were filtered out based on the mean-variance trend of the data. The expected decreasing trend between data mean and variance was observed when expressed transcripts were determined as ≥ 1 of the 14 samples with count per million reads (CPM) ≥ 4, which provided an optimal filter of low expression. A gene was declared to be expressed if any of its transcripts met the above criteria. The TMM method was used to normalize the gene and transcript read counts to log_2_-CPM, and a principal component analysis (PCA) plot showed that the RNA-seq data did not have distinct batch effects. The log_2_ fold change (FC) of gene/transcript abundances was calculated based on contrast groups and the significance of expression changes was determined using t-test. A gene/transcript was significantly differentially expressed (DE) in a contrast group if it had an absolute log_2_ FC ≥ 1 and an adjusted *P*-value < 0.01 after applying multiple testing (Benjamini et al., 2001). For comparison with Kim et al. (2017) data an adjusted *P*-value of < 0.05 was used. At the alternative splicing level, DTU (differential transcript usage) transcripts were identified by comparing the log_2_ FC of a transcript to the weighted average of log_2_ FCs (weights were based on their standard deviation) of all remaining transcripts of the same gene. A transcript was declared to exhibit significant DTU if it had an adjusted *P*-value < 0.01 and a Δ Percent Spliced (ΔPS) ratio ≥ 0.1. For DAS genes, each individual transcript log_2_ FC was compared to the gene-level log_2_ FC, which was calculated as the weighted average of log_2_ FCs of all transcripts of the gene. Then *P*-values of individual transcript comparison were summarized to a single gene-level *P*-value with an F-test. A gene was significantly DAS in a contrast group if it had an adjusted *P*-value < 0.01 and any of its transcripts had a ΔPS ratio ≥ 0.1.

Transcript isoform switches (ISs) were recognized as such if the order of relative expression levels of a pair of alternatively spliced isoforms underwent a reversal. The Pair-Wise Isoform Switch (isokTSP) method was used to detect the isoform switch points between conditions of contrast groups (except for the 6-days samples). The method defined the ISs between any pair of transcripts within genes using mean values of conditions. It described the significant ISs using five different features of metrics: 1) the probability of switching (i.e. the frequency of samples reversing their relative abundances at the switches) was set to > 0.5; (2) the sum of the average differences of the two isoforms in both intervals before and after the switch point was set to ΔTPM > 1; (3) the significance of the differences between the switched isoform abundances before and after the switch was set to a BH-adjusted *P*-value < 0.01; (4) both of the interval lengths before and after switch were set to 1; and (5) the Pearson correlation of two isoforms was set to >0 (see Guo et al. (2021) for more details).

### 2.7 Visualization of gene expression data

Normalized read depths of transcripts were visualized with the Integrative Genomics Viewer (IGV; Robinson et al., 2011). The heatmaps were generated with the help of ClustVis, a web tool for visualizing clustering of multivariate data (Metsalu and Vilo, 2015).

### 2.8 Determination of gene ontology enrichments

GO enrichments were obtained from the Database for Annotation, Visualization and Integrated Discovery (DAVID; Huang da et al., 2009), applying a cut-off of 2-fold enrichment compared to the expected frequency in the Arabidopsis genome and an FDR (Benjamini-Hochberg) ≤ 0.05. Non-redundant GO terms were selected in the interface REVIGO using the medium similarity (0.7) parameter (Supek et al., 2011).

## 3 Results

### 3.1 Drought-related changes in gene expression are robust across different laboratories

**Figure 1:**
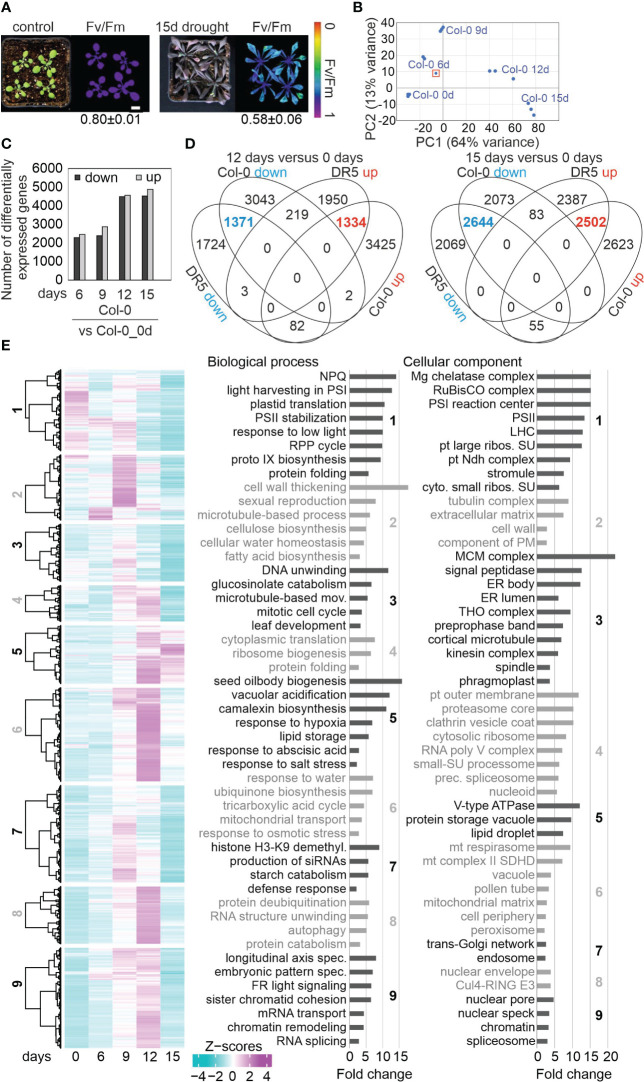
Drought-related changes in gene expression are robust across different laboratories. **(A)** Phenotypic characterization of Col-0 plants grown for 3 weeks under normal, well-watered, conditions (control), and then subjected to drought stress by withholding water for 15 days. The maximum quantum efficiency of photosystem II (Fv/Fm) was measured with an imaging Chl fluorometer (Imaging PAM). Bar = 1 cm. **(B)** Principal Component Analysis (PCA) plot visualizing variation between replicates and time-points based on RNA-seq data. The sample marked with a red rectangle was not used for further analysis. **(C)** Numbers of differentially expressed genes (absolute log_2_ fold change ≥1; in at least one contrast group with an adjusted *P* < 0.01) subjected to drought exposure compared to the control (0 days) at the indicated time points. **(D)** Comparison of transcript changes evoked by drought stress in Col-0 (this study) or in DR5 [re-analyzed data from Kim et al. (2017)]. Venn diagrams illustrate the total numbers of differentially expressed genes shared between or specific for the different treatments. **(E)** Overview and gene ontology analysis of gene expression changes under drought. Heatmap of differentially expressed (DE) genes under drought stress compared to the control time point (0 days). Hierarchical clustering was used to partition the DE genes into nine clusters with the Euclidean distance and ward.D clustering algorithm. Right side: Graphs illustrating non-redundant Gene Ontology (GO) term enrichment for the biological process and cellular component categories according to DAVID (Huang da et al., 2009) and REVIGO (Supek et al., 2011). GO terms with a >2-fold change and a Benjamini-corrected *P*-value of <0.05 are shown. Cul4-RING E3, Cul4-RING E3 ubiquitin ligase; LHC, light-harvesting complex; mov., movement; NPQ, nonphotochemical quenching; prec., precatalytic; protein catabolism, ubiquitin-dependent protein catabolism; RPP, reductive pentose-phosphate; spec., specification; PS, photosystem; pt, plastid; ribos., ribosomal; SU, subunit.

Our goal was to investigate in addition to differential gene expression, the behavior of post-transcriptional processing of nuclear or organellar transcripts under drought. To this end, RNA sequencing was performed with ribosomal RNA-depleted RNA isolated from 3-week-old Col-0 plants grown under optimal conditions (time-point 0 days; 0d_DS), and after withholding of water for 6, 9, 12 and 15 days (6d_DS, 9d_DS, 12d_DS and 15d_DS, respectively) as described previously (Kim et al., 2017). At 15d_DS, Col-0 accumulated anthocyanins, wilted and displayed lower maximum quantum yield of photosystem (PS) II (Fv/Fm; **Figure 1A**). Here it is of note, that Kim et al. (2017) used microarrays, which are not suitable for our purpose – monitoring of organellar transcript accumulation and editing events, and the investigation of splicing of transcripts produced in organelles as well as the nucleus. We repeated our experiment separately with different batches of plants, yielding three biological repeats per time point. Principal-component analysis showed that drought was an important driver of gene expression and replicates clustered together (**Figure 1B**). One replicate of 6d_DS was an outlier and the sequencing quality did not meet our criteria and only two replicates were used for further analysis. The sequencing depth was approximately 25 million 150-bp paired-end reads for each of the samples (**Supplementary Table 2**).

Investigation of whole-genome wide gene expression changes showed that 16,850 genes were considered as expressed after removal of weakly expressed ones, and most of them (12,523) were differentially expressed in response to drought when compared with the starting condition (**Figure 1C**; **Supplementary Table 3**). These findings again show that drought has a substantial impact on the transcriptome. Here, a gene was considered to be differentially expressed (DEG) if it showed an absolute log_2_ fold change ≥1 (≥ 2-fold linear change) in expression in at least one contrast group (adjusted *P* < 0.05). 49.2% of these were down- and 50.8% upregulated, respectively (**Figure 1C**). Our re-analysis of data published by Kim et al. (2017), and comparison with the transcriptome data generated in the present study, showed that drought-related changes in gene expression are robust across different laboratories and transcriptome platforms after prolonged exposure to drought stress (**Figure 1D**). Accordingly, we provide lists of robust drought-responsive DEGs in **Supplementary Table 4**. Some of these were exceedingly differentially expressed. For example, levels of *AT1G66100* mRNA were reduced to 0.005% of control levels after 15 days (**Supplementary Table 3**).

### 3.2 Gene ontology analysis

**Figure 2:**
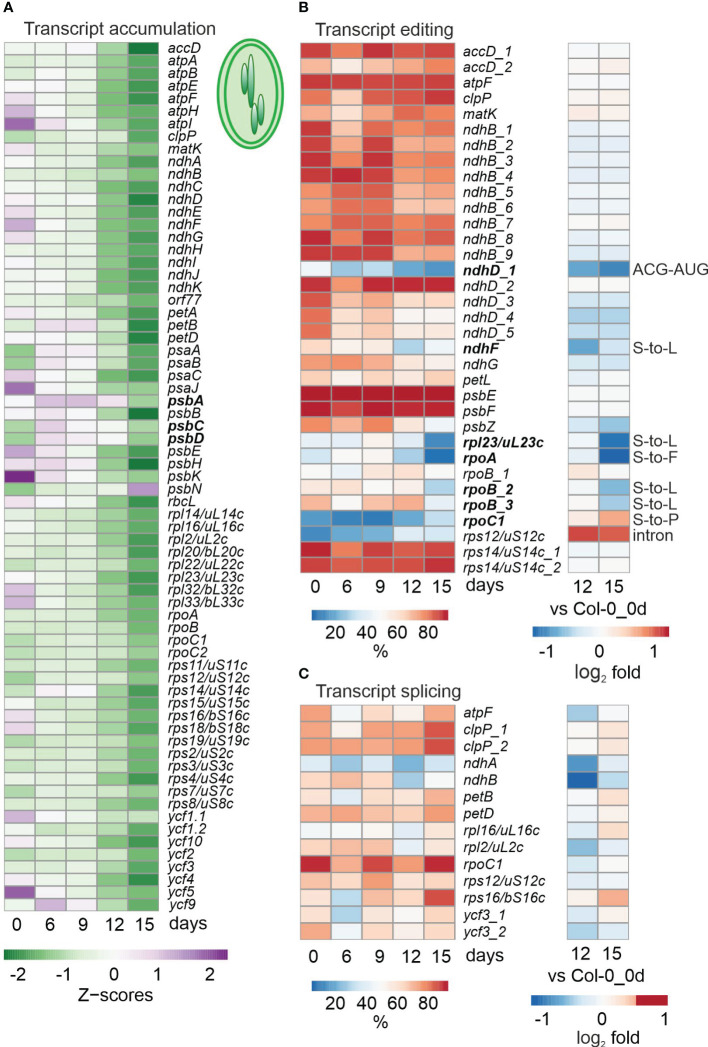
Impact of drought treatment on accumulation, editing and splicing of chloroplast transcripts. Water was withheld for 15 days from 3-week-old Col-0 plants grown under standard conditions (0 days), and RNA-Seq was performed as described in Materials and Methods on RNA extracted from plants harvested at the indicated time-points. **(A)** Heatmap illustrating chloroplast transcript accumulation (Z-scores) during the drought time-course. Low to high expression is represented by the green to purple transition. Note that Z-scores are calculated for each individual transcript over the time course. **(B, C)** Percentages of editing **(B)** and splicing **(C)** events during the time-course (left) and log_2_ fold changes after 12 and 15 days of drought stress compared to the control time point (right). The effect of editing changes is shown in panel **(B)** on the right.

**Figure 3:**
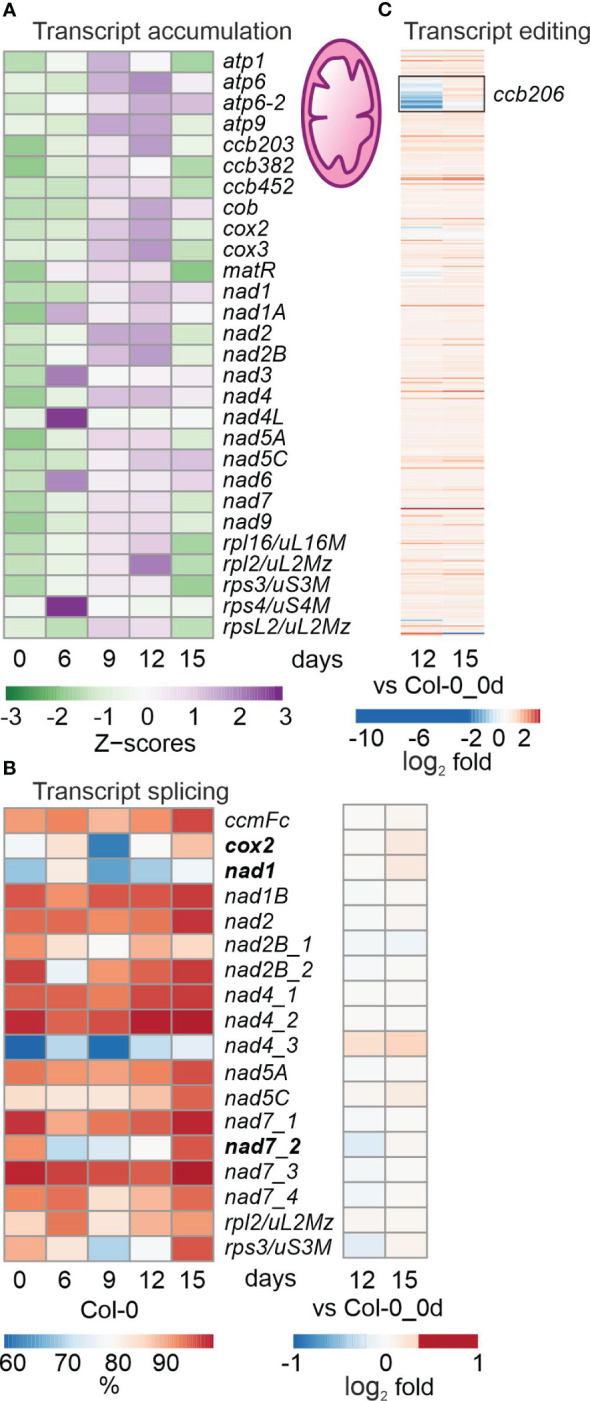
Impact of drought treatment on the accumulation, editing and splicing of mitochondrial transcripts. Plants and RNA were treated and analyzed, and data are depicted as described in the legend to **Figure 2**. **(A)** Heatmap illustrating chloroplast transcript accumulation (Z-scores) during the drought time-course. **(B)** Percentages of transcript splicing during the time course (left) and log_2_ fold changes after 12 and 15 days of drought stress compared to the control time point (right side). **(C)** Log_2_ fold changes of transcript editing during the time course.

The kinetic data were further explored by sorting the DEGs into nine different clusters. Cluster 1 contained the genes that were strongly downregulated at 12d_DS and 15d_DS. Gene Ontology (GO) analysis of this cluster showed enrichments of genes encoding proteins related to the response to low light, protein folding, chlorophyll biosynthesis, cp translation and photosynthesis-related processes in the biological process (BP) category (**Figure 1E**; **Supplementary Table 5**). This is in line with the phenotype and Fv/Fm value of Col-0 under drought. Moreover, the GO analysis revealed that, in addition to chloroplast (cp)-encoded genes (see **Figure 2**), nucleus-encoded chloroplast proteins are also regulated at the transcript level. In the cellular component (CC) category, transcripts coding for components of the PSI, PSII and the cp Ndh complexes, as well as cp and cytosolic ribosomal subunits, were enriched (**Figure 1E**). The similarities in the behavior of transcripts for both cp and cytosolic ribosomal subunits reflect the importance of coordination between protein synthesis in chloroplasts and the cytosol (Wang R. et al., 2018) during drought stress. The majority of mt transcripts in Col-0 increased or barely changed under drought (see **Figure 3**). Accordingly, genes encoding mt proteins were found in cluster 6, which encompasses genes whose transcript levels especially increased at 9d_DS and 12d_DS, in particular those encoding proteins of the substrate-carrier family, succinate-dehydrogenase complex II and enzymes of the tricarboxylic acid cycle (TCA). Indeed, TCA cycle metabolites are known to increase after water shortage (Pires et al., 2016). Cluster 6 also includes the categories “peroxisome”, “response to water” and “response to osmotic stress”. The latter two categories both point to a disturbance in water balance, and genes encoding proteins involved in responses to hypoxia, abscisic acid and salt stress are found in cluster 5, which encompasses genes that behave like those of cluster 6. Moreover, genes assigned to the GO categories “cellular water homeostasis”, together with “cell wall thickening” and “fatty acid biosynthesis” in cluster 2 are specifically induced after 6 or 9 days of drought treatment.

In summary, drought treatment induces massive transcriptional reprogramming, and the polarities of changes in gene expression (up or down) are compatible with responses to drought at the phenotypical and metabolic levels. In addition, mRNA expression of the organellar and nuclear genomes are coordinated.

### 3.3 Drought stress has a negative impact on the chloroplast (post)transcriptome

Heat stress for periods of several hours induces a global reduction in splicing and editing efficiency in chloroplasts (cp), and an overall increase in the abundance of cp transcripts, while short-term drought stress for 3 and 12 hours results in only minor changes in cp transcripts (Castandet et al., 2016).

To investigate cp transcripts under drought stress, reads were mapped and processed with the help of ChloroSeq (Castandet et al., 2016). Overall levels of noncoding cp RNAs are higher after 12 h of heat treatment (Castandet et al., 2016), but inspection of our bamcoverage files, which include reads across all nucleotide (nt) positions of the cp genome, showed that this does not hold for exposure to drought (**Supplementary Figure 1**). In fact, cp coverage already indicated lower overall cp transcript accumulation in Col-0 after prolonged drought (**Supplementary Figure 1**). To investigate this for individual transcripts, heatmaps of z-means (**Figure 2A**) and fold changes of cp transcripts (**Supplementary Table 6A**) were generated, which revealed substantial reductions in levels of most transcripts under prolonged drought conditions in Col-0 (**Figure 2A**; **Supplementary Table 6A**). Here, it should be noted that analysis of tRNAs was excluded (also in the following editing and splice-site analyses) because their small size and many modifications make them difficult to amplify with either our RNA-Seq method or conventional mRNA-Seq protocols. Of the 69 protein-coding cp genes that were above the detection threshold, 50, 63 and 64 were at least 2-fold reduced (compared to the 0d_DS sample) in Col-0 plants after 9, 12 and 15 days of drought, respectively. The genes most affected (those whose transcripts were reduced to 3% or less of their initial levels) code for the D3, F and K subunits of the NAD(P)H dehydrogenase complex and a protein of the large ribosomal subunit (rpl23, uL23c according to the Nomenclature of Ribosomal Proteins; https://bangroup.ethz.ch/research/nomenclature-of-ribosomal-proteins.html and Scarpin et al., 2023).

To examine post-transcriptional changes in cp transcripts, editing and splicing efficiencies were calculated. Interestingly, unlike transcript accumulation, editing was not generally reduced in Col-0 under drought conditions, but was observed especially for *ndhD*, *ndhF*, *rpl23*/*uL23c* and the *rpoA* and *rpoB* genes encoding α and β subunits of plastid-encoded RNA polymerase (**Figure 2B**). Although these editing changes do not result in premature stop codons, but rather amino acid changes or an alternative start codon, they may alter the structure of RNA or proteins, or affect the ability of altered proteins to form complexes with other proteins. Moreover, editing of *rps12* and *rpoC1* were enhanced and slightly enhanced, respectively. After 15 days of drought treatment, splicing efficiency was not notably diminished, and in some cases (five out of 14 events) was slightly enhanced (**Figure 2C**).

To summarize, while overall cp mRNA levels were broadly reduced, impairment of editing events was more marked, and splicing was not compromised at all.

### 3.4 Mitochondrial transcript accumulation, editing and splicing under drought

Mitochondrial (mt) transcripts were also investigated, and inspection of bamcoverage files of the mt genome suggested even higher mt transcript accumulation under drought (**Supplementary Figure 2**), in contrast to the fall in levels of cp transcripts (see **Supplementary Figure 1**). This was confirmed by producing heatmaps of z-means of mt transcripts and their fold changes (**Figure 3A**; **Supplementary Table 6B**). Thus, levels of the majority of mt mRNAs increased or were only slightly changed during drought treatment (**Figure 3A**; **Supplementary Table 6B**). Of the 49 protein-coding mt genes that were above the detection threshold, 11, 11 and 26 transcripts were at least 2-fold elevated after 9, 12 and 15 days of drought, respectively, compared to 0d_DS. Only a few mRNAs were down-regulated during drought stress, of which the two most strongly reduced transcripts *ATP synthase subunit 1* (*atp1*; 0.3%) and *rpsL2* (*ATMG00980*; 17.8%) were detected at 15d_DS. Interestingly, transcripts coding for mt NADH dehydrogenase subunits increased during prolonged drought treatment – in stark contrast to their counterparts in the cp sister complex.

With regard to post-transcriptional changes of mt transcripts, transient down-regulation of splicing efficiency was observed for *cytochrome oxidase 2* (*cox2*), *nad1* and *nad7-2*, and splicing capacity was slightly enhanced after 15 days (**Figure 3B**). Editing capacity under drought was not changed relative to 0d_DS, except in the case of *ccb206* (encoding the cytochrome *c* biogenesis protein 206), which was reduced in Col-0 at 12d_DS (**Figure 3C**).

Overall, mt (post)transcription was not markedly impaired and drought-induced changes tended to show the opposite trend to that seen in the cp transcripts. However, it should be noted that the level of *atp1* transcripts was reduced to 0.3% after 15 days of drought stress.

### 3.5 Splicing of nuclear transcripts under drought stress

**Figure 4:**
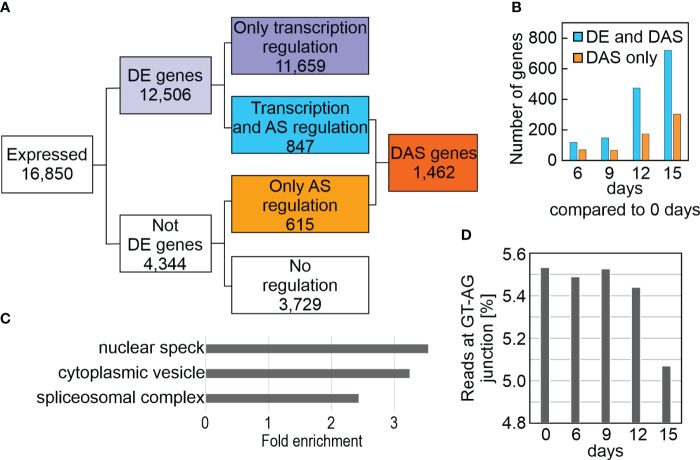
Impact of drought treatment on differential alternative splicing (DAS). DAS was calculated as described in Materials and Methods. **(A)** Flow chart showing the distribution of differentially expressed (DE) and DAS genes. **(B)** Number of DE and DAS genes in Col-0 plants exposed to drought stress compared to the control condition (0 days). **(C)** GO enrichment analysis of the cellular component category according to DAVID (Huang da et al., 2009). GO terms with a >2-fold change and a Benjamini corrected value of <0.05 are shown. **(D)** Percentage of reads spanning the canonical GT-AG splice junction in Col-0 under control (0 days) and drought conditions.

To systematically investigate splicing behavior under drought stress, genome-wide differential alternative splicing (DAS) and differential transcript usage (DTU) during drought stress was examined with the help of the high-quality AtRTD2_QUALI transcriptome and 3D RNA-Seq (Zhang et al., 2017; Guo et al., 2021). By this means, in addition to the expression at the gene level (i.e., the sum of all transcript abundances of a given gene), the individual transcript isoform levels could be determined (**Supplementary Table 7**). In all, 1,462 DAS genes were identified, of which 847 were also DE genes (regulated by both transcription and AS), while 615 genes were regulated by only AS in at least one contrast group (**Figure 4A**; **Supplementary Table 8**). Among the latter are LESION SIMULATING DISEASE 1 (LSD1), which monitors a superoxide-dependent signal and negatively regulates plant cell death (Bernacki et al., 2021), as well as playing an important role in survival of drought stress (Szechynska-Hebda et al., 2016), plastidic acetyl-CoA synthetase (ACS), REGULATORY PARTICLE TRIPLE-A ATPASE 6A (RPT6A) which is a component of the 26S proteasome AAA-ATPase subunit, PROTEASOME REGULATOR1 (PTRE1), and protein synthesis initiation factor eIF2 beta (**Supplementary Table 8**). Notably, DAS increased after prolonged drought stress (**Figure 4B**), and was especially pronounced for *GLYCINE RICH PROTEIN 7* (*ATGRP7*). Differential splicing of transcripts of the three phytochrome genes *PHYB*, *PHYC* and *PHYE*, together with *PHYTOCHROME INTERACTING FACTOR 3* (*PIF3*), *PIF3-LIKE 5* and *6*, and *FYPP3* (coding for phy-associated protein phosphatase 3) is particularly striking. PhyB acts in multiple environmental stress pathways (Kim et al., 2021), and PhyB positively mediates drought tolerance (Gonzalez et al., 2012). In summary, 9% of the 16,850 expressed genes were differentially alternatively spliced and 3,283 transcript isoforms were detected whose splicing patterns were altered in at least one contrast group. GO analysis of the corresponding loci revealed enrichments of transcripts for proteins that were localized to the nuclear speck, cytoplasmic vesicle (for example, the AUTOPHAGY group of ubiquitin-like superfamily proteins – ATG8B, E and F, and ATG13) and the spliceosomal complex itself (**Figure 4C**). Indeed, this is supported by a reduction in the percentage of reads covering the canonical GT-AG splice junction (**Figure 4D**).

These results suggest the importance of alternative splicing of nuclear genes in the modulation of responses to drought stress.

### 3.6 Isoform switch analysis – usage of different isoforms during the course of drought stress

**Figure 5:**
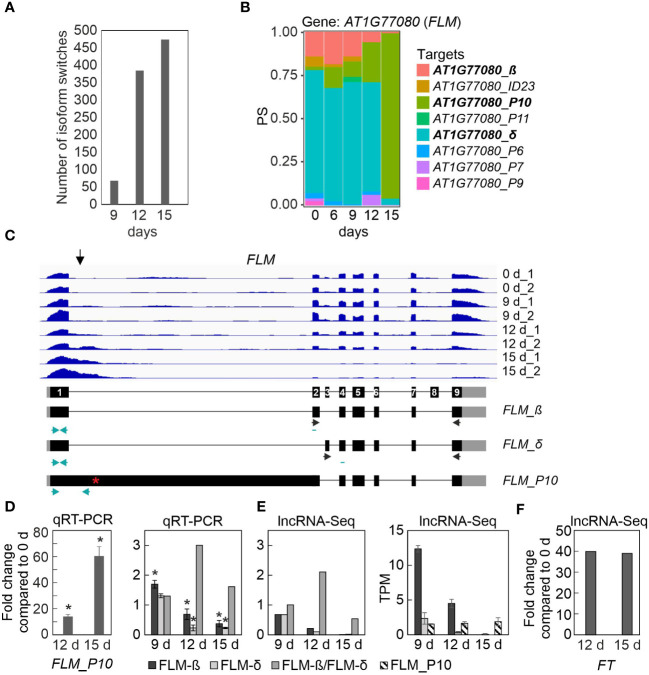
Transcript isoform switch (IS) analysis uncovers different *FLOWERING LOCUS M* (*FLM*) isoforms. IS were calculated as described in Materials and Methods. **(A)** Numbers of ISs detected in Col-0 after 9, 12 and 15 days of drought stress. **(B)** Expression profiles of *FLOWERING LOCUS M* (*FLM*) at the whole-gene level (*AT1G77080*) and at the level of detected transcript isoforms are shown. PS, percentage of expressed transcripts spliced. **(C)** Pattern of *FLM* transcript accumulation. The normalized read depths of transcripts detected in Col-0 plants under control and prolonged drought conditions were visualized with the Integrative Genomics Viewer (IGV). The vertical arrow indicates the most differentially spliced region. Exons (black boxes), introns (black lines) and the 5′- and 3′-UTRs (gray boxes) are shown. Black and turquoise horizontal arrows indicate the positions of primers used in RT-PCR reactions done in previous publications (e.g. Pose et al., 2013) and in this publication, respectively. Note that the primers used to amplify *FLM-β* and *FLM-δ* isoforms are exon-exon spanning. Red asterisk, premature stop codon. **(D)** Quantitative reverse transcriptase-polymerase chain reaction (qRT-PCR) analysis of Col-0 control plants (0 d) and plants subjected to drought stress for 12 and 15 days (12 d and 15 d). qRT-PCR was performed with primers specific for the FLM_P10, *FLM-β* and *FLM-δ* isoforms as indicated by the turquoise arrows in panel **(C)**, and for *AT3G58500* [encoding PROTEIN PHOSPHATASE 2A-4 (PP2A-4)], which served as a control because it did not significantly change its expression level under drought. Expression values are reported relative to the corresponding transcript levels in non-stressed Col-0. The results were normalized with respect to the expression level of *PP2A-4*. Bars indicate standard deviations. Statistically significant differences (*P* < 0.05) between stressed and non-stressed samples are indicated by an asterisk. Note that the *FLM-β* primers probably detect non-canonical splice forms, in addition to *FLM-β* (Sureshkumar et al., 2016). **(E)** Fold changes and TPM (Transcripts Per Million) values of FLM isoforms. **(F)** Fold changes of FT transcripts relative to Col-0 control plants (0 d). Data were extracted from RNA-Seq (lncRNA-Seq) results.

We then sought to identify DAS genes that showed isoform switches (ISs), i.e. in which the relative abundance of different isoforms was altered over the course of drought exposure. In total, 927 (covering 404 gene loci; *P* < 0.01) ISs that involved abundant transcript isoforms were detected (**Figure 5A**; **Supplementary Table 9**). The majority of ISs (more than 450) were identified after 15 days of drought stress. Among the transcripts undergoing ISs were the above-mentioned *LSD1* and *PHYB*. Other examples include *CARBAMOYL PHOSPHATE SYNTHETASE B* (*CARB*, also named *VENOSA 3*), *ZINC-INDUCED FACILITATOR 2* (*ZIF2*), pre-mRNA processing *PRP39A*, and *FLOWERING LOCUS M* (*FLM*) (**Figure 5B**; **Supplementary Figure 3**). The ISs involved different types of AS events, which either generated isoforms that encoded different protein variants, or occurred in the 5′ or 3′UTR, without changing the sequence of the protein (**Supplementary Table 9**). IS events were also detected in transcripts that did not encode a functional protein, e.g., they contained a premature stop codon or did not contain the start codon (**Supplementary Table 9**). For example, the s3 isoform of the *CARB* transcript that accumulated under prolonged drought stress is predicted not to be translated, as is the P5 isoform of *PRP39A*, and the predicted s3 isoform is very short (**Supplementary Figure 3**).

In conclusion, RNA-Seq analysis uncovered numerous DAS and IS events, including the accumulation of putative non-functional transcript isoforms.

### 3.7 Suggested candidates for further investigation and proof of concepts

We propose in **Table 1** several potential candidates from different categories (DE genes, DE transcripts, DAS, IS events, chloroplast and mitochondrial transcripts) for further investigation of responses to drought stress. For example, we include specific genes encoding proteins involved in defense responses; pathogenesis-associated proteins and peptides have already been proposed as promising tools for developing plants with multiple stress tolerance (Ali et al., 2018).

**Table 1:** List of suggested candidates for further investigation of their involvement in drought stress responses.

Differentially expressed genes (see Supplementary Table 3; regulated in both our dataset as well as in Kim et al., 2017)				
Gene ID	Protein Description	Gene Symbol	log2 12d	log2 15d
*AT1G66100*	Predicted to encode a PR protein. Belongs to the plant thionin (PR-13) family†		-12,5	-11,3
*AT5G36910*	Predicted to encode a PR protein. Belongs to the plant thionin (PR-13) family†	*THI2.2*	-9,2	-6,6
*AT3G05730*	Defensin-like (DEFL) family protein		-11,7	-10,4
*AT5G65730*	Hydrolase activity, acting on glycosyl bonds, involved in response to water deprivation	*XTH6*	-11,4	-9,2
*AT4G04840*	METHIONINE SULFOXIDE REDUCTASE B6, has peptide-methionine-(S)-S-oxide reductase activity	*MSRB6*	-11,1	-5,6
*AT2G18300*	HOMOLOG OF BEE2 INTERACTING WITH IBH 1; basic helix-loop-helix (bHLH) DNA-binding superfamily protein	*HBI1*	-10,5	-9,3
*AT1G03870*	FASCICLIN-LIKE ARABINOOGALACTAN 9	*FLA9*	-10,5	-9,3
*AT5G44020*	HAD superfamily, subfamily IIIB acid phosphatase		-10,2	-10,8
*AT2G21650*	MATERNAL EFFECT EMBRYO ARREST 3; member of a small sub-family of single MYB transcription factors	*MEE3; RSM1*	-9,8	-9,1
*AT2G29290*	NAD(P)-binding Rossmann-fold superfamily protein; functions in oxidoreductase activity		-9,6	-8,4
*AT1G72610*	GERMIN-LIKE PROTEIN 1	*GER1; GLP1*	-9,5	-8,2
*AT5G48490*	Protein with similarity to a lipid transfer protein that may contribute to systemic acquired resistance (SAR)	*DIR1-LIKE*	-9,5	-7,0
*AT3G01500*	CARBONIC ANHYDRASE 1, regulates together with betaCA4 (At1g70410) CO2-controlled stomatal movements in guard cells, see also main text and **Figure 6**	*CA1*	-8,4	-10,8
*AT5G52300*	RESPONSIVE TO DESICCATION 29B; induced in expression in response to water deprivation such as cold, high-salt, and desiccation	*RD29B; LTI65*	11,3	10,9
*AT4G12960*	Gamma interferon responsive lysosomal thiol (GILT) reductase family protein		11,2	9,8
*AT1G61800*	GLUCOSE-6-PHOSPHATE/PHOSPHATE TRANSLOCATOR 2	*GPT2*	9,7	10,1
*AT2G29380*	HIGHLY ABA-INDUCED PP2C GENE 3, protein serine/threonine phosphatase activity	*HAI3*	9,3	8,6
Differentially expressed transcript isoforms (see Supplementary Table 7)				
**Transcript ID**	**Protein Description**	**Gene Symbol**	**log2 12d**	**log2 15d**
*AT3G14210_s1*	EPITHIOSPECIFIER MODIFIER 1; represses nitrile formation and favors isothiocyanate production; functional allele deters the insect herbivory *T. ni*.	*ESM1*	-6,7	-12,6
*AT3G01500_ID11*	See above	*CA1*	-8,1	-11,6
*AT3G01500.2*	See above	*CA1*	-11,8	-11,1
*AT4G26530.2*	FRUCTOSE-BISPHOSPHATE ALDOLASE 5; involved in glycolysis	*FBA5*	-10,1	-11,2
*AT4G26530_P3*	FRUCTOSE-BISPHOSPHATE ALDOLASE 5	*FBA5*	-7,4	-9,5
*AT5G44020.1*	HAD superfamily, subfamily IIIB acid phosphatase		-10,2	-10,8
*AT4G14400_P1*	ACCELERATED CELL DEATH 6; member of the largest uncharacterized gene families in higher plants; involved in resistance to *Pseudomonas syringae*	*ACD6*	-9,7	-9,9
*AT1G75600_P1*	Histone superfamily protein; involved in nucleosome assembly	*HTR14*	7,4	11,0
*AT3G01420_P1*	PLANT ALPHA DIOXYGENASE 1; involved in protection against oxidative stress and cell death	*PADOX-1; DOX1*	9,1	10,0
*AT1G32350_P1*	ALTERNATIVE OXIDASE 1D; mitochondrion	*AOX1D*	8,0	9,9
*AT5G52300_P1*	See above	*RD29B*	11,4	10,8
*AT4G33150_P4*	Encodes two proteins. One protein is the monofunctional saccharopine dehydrogenase involved in lysine degradation. The longer protein from the same LKR/SDH locus is bifunctional and also has saccharopine dehydrogenase activity. Gene expression is induced by ABA, jasmonate, and under sucrose starvation		8,3	10,1
*AT2G27150.2*	ABSCISIC ALDEHYDE OXIDASE 3; aldehyde oxidase delta isoform catalyzing the final step in ABA biosynthesis‡	*AAO3*	9,5	9,1
Differentially alternatively spliced, but not differentially expressed (see Supplementary Table 8)				
**Gene ID**	**Protein description**	**Gene symbol**		
*AT4G20380*	LESION SIMULATING DISEASE 1; monitors a superoxide-dependent signal and negatively regulates a plant cell death pathway	*LSD1*		
*AT5G19990*	REGULATORY PARTICLE TRIPLE-A ATPASE 6A; 26S proteasome AAA-ATPase subunit	*RPT6A*		
*AT3G53970*	PROTEASOME REGULATOR 1; was identified as homologous to human PI31	*PTRE1*		
*AT2G18790*	PHYTOCHROME B; red/far-red photoreceptor	*PHYB*		
*AT1G09530*	PHYTOCHROME INTERACTING FACTOR 3	*PIF3*		
*AT2G20180*	PIF3-LIKE 5; myc-related bHLH transcription factor	*PIL5*		
*AT3G59060*	PIF3-LIKE 6; myc-related bHLH transcription factor	*PIL6*		
*AT3G19980*	Phy-associated protein phosphatase 3	*FYPP3*		
*AT4G04620, AT2G45170, AT4G16520, AT3G49590*	AUTOPHAGY group of ubiquitin-like superfamily proteins	*ATG8B, ATG8E, ATG8 F, ATG13*		
*AT1G21980*	PHOSPHATIDYLINOSITOL-4-PHOSPHATE 5-KINASE 1; preferentially phosphorylates PtdIns4P. Induced by water stress and ABA	*PIP5K1*		
*AT3G44850*	Protein kinase superfamily protein			
*AT4G15010*	Mitochondrial substrate carrier family protein			
*AT3G60910*	S-adenosyl-L-methionine-dependent methyltransferases superfamily protein			
*AT4G32140*	EamA-like transporter family protein			
Isoform switch (see Supplementary Table 9)				
**Gene ID**	**Protein description**	**Gene symbol**		
*AT4G20380*	see above	*LSD1*		
*AT2G18790*	see above	*PHYB*		
*AT1G29900*	CARBAMOYL PHOSPHATE SYNTHETASE B	*CARB; VEN3*		
*AT2G48020*	ZINC-INDUCED FACILITATOR 2	*ZIF2*		
*AT1G04080*	Pre-mRNA processing protein	*PRP39A*		
*AT1G77080*	FLOWERING LOCUS M; MADS domain protein; flowering regulator that is closely related to FLC; see also main text	*FLM*		
*AT1G09140*	SERINE/ARGININE-RICH PROTEIN SPLICING FACTOR 30	*SR30; SRP30*		
Chloroplast-encoded transcripts (Supplementary Table 6A)				
Genes with most down-regulated transcripts encode D3, F and K subunits of the NAD(P)H dehydrogenase complex and a protein of the large ribosomal subunit (rpl23/*uL23c*)				
Edititing altered for *ndhD*, *ndhF*, *rpl23*/*uL23c* and the *rpoA* and *rpoB* genes encoding α and β subunits of plastid-encoded RNA polymerase				
Mitochondrial-encoded transcripts (Supplementary Table 6B)				
Reduced transcripts for ATP synthase subunit 1 (atp1; 0.3%) and rpsL2 (17.8%) at 15d_DS. Interestingly, transcripts coding for mt NADH dehydrogenase subunits increased during prolonged drought stress – in stark contrast to their counterparts in the cp sister complex				

†Pathogenesis-related proteins and peptides as promising tools for engineering plants with multiple stress tolerance (Ali et al., 2018), ‡involved in drought stress, investigated in rice (Shi et al., 2021).

**Figure 6:**
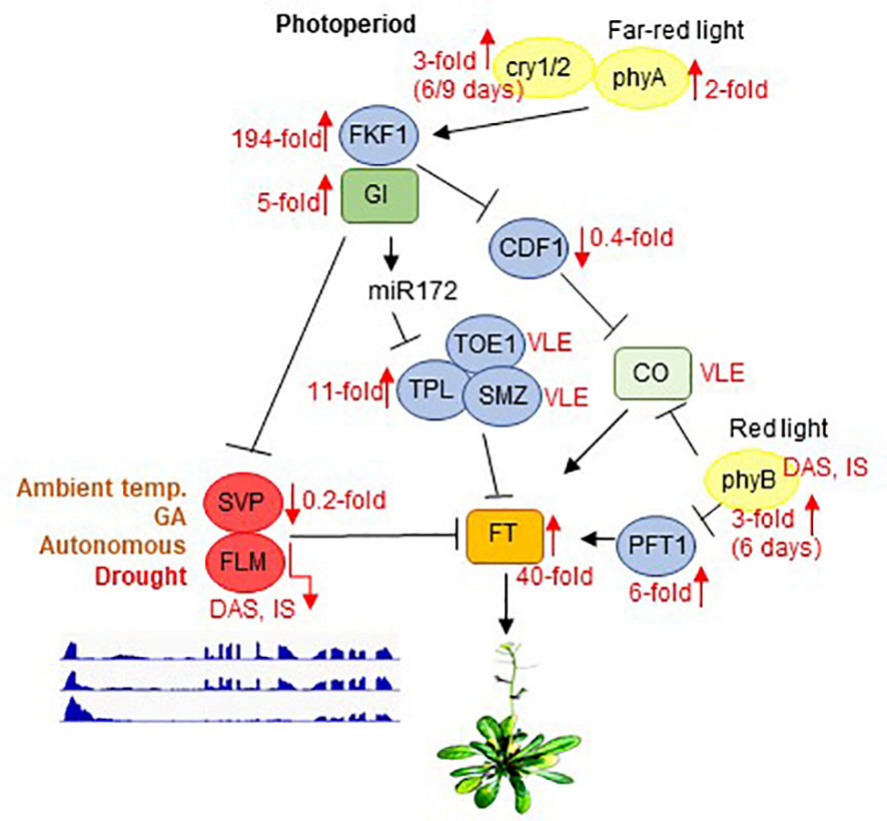
Scheme depicting the photoperiod-dependent flowering pathway, together with inputs from light receptors. Note that the components of the flowering pathway are mainly regulated through transcriptional activation or repression. SVP and FLM are components of the ambient-temperature, GA and autonomous pathways. GI acts in both CO-dependent (by suppressing CDF1) and CO-independent branches by either repressing SVP or by promoting *miR172* expression to control flowering. Flowering itself is ultimately mediated by FT, which is an inducer of flowering. phyB acts to suppress CO protein activity, whereas phyA, cry1, and cry2 function to enhance the activity of CO. In parallel, phyB affects *FT* transcription by suppressing *PFT1*, an upstream activator of *FT*. Black arrows and T-ends indicate positive or negative regulation, respectively. Red arrows together with numbers indicate fold changes after drought stress. *FLM* and *PHYB* transcripts are subject to DAS and IS. Only the main regulatory genes are shown here. The complete flowering time network involves several hundred genes, and is available on the WikiPathways website (https://www.wikipathways.org/index.php/Pathway: WP2312). Modified after Kim (2020) and Leijten et al. (2018). DAS, differential alternative splicing; IS, isoform switching; temp., temperature; VLE, very low expressed.

Two of the candidates listed in **Table 1** were selected for proof-of-concept studies. First, the expression behavior of the *FLM* transcript isoforms is particularly noteworthy. As shown by analysis of RNA-Seq data (**Figures 5B, C**) and confirmatory qRT-PCR (**Figure 5D**), the P10 isoform accumulated to a very high level (15-fold and 60-fold after 12 and 15d_DS, respectively, compared with 0d_DS) during the drought treatment in Col-0 at the expense of *FLM-β* and *FLM-δ* isoforms (**Figure 5E**), which was confirmed by qRT-PCR (**Figure 5E**). The P10 form covers the first exon and part of the first intron (**Figure 5C**), resulting in a premature stop codon (**Supplementary Table 9**). It has been suggested that *FLM-β* is the functional protein that can prevent early flowering by repressing the expression of the key flowering time regulator FLOWERING LOCUS T (FT, florigen) (summarized in: Zarnack et al. (2020) and Quiroz et al. (2021)). We observed a 40-fold induction of *FT* transcript levels after 12 and 15 days of drought stress compared with 0d_DS (**Figure 5F**), but after 15 days of drought treatment, *FLM-β* and *-δ* expression levels were almost undetectable, suggesting that the absence of FLM protein is sufficient to mediate the early flowering phenotype under drought conditions (see **Figure 6**, Discussion section).

**Figure 7:**
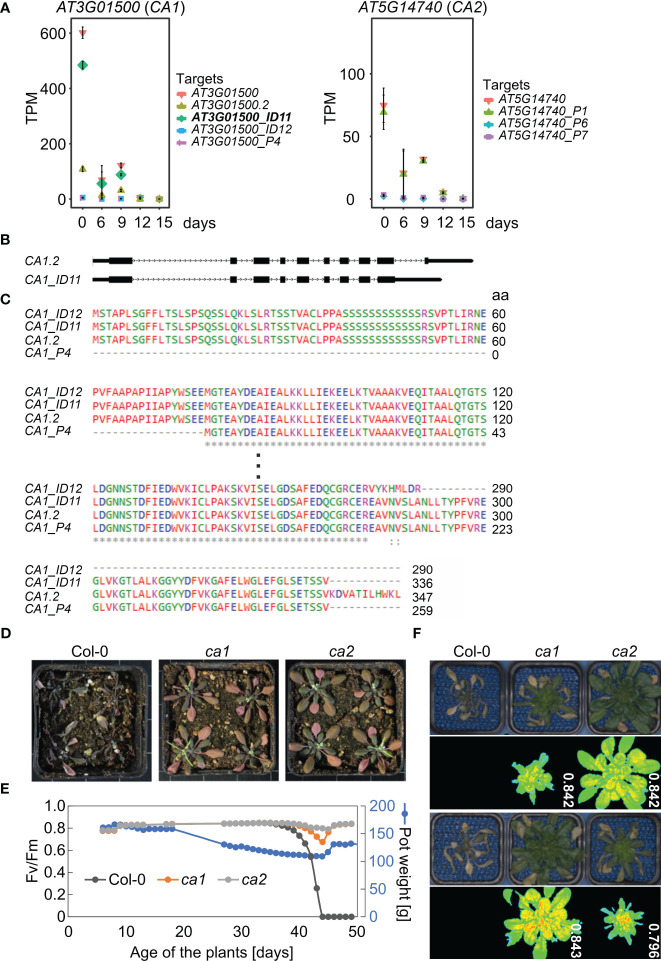
Carbonic anhydrases CA1 and CA2 are involved in drought tolerance. **(A)** Expression profiles of *CA1* and *CA2* at the whole-gene level and at the level of detected transcript isoforms. TPM, transcripts per million reads. **(B)** Scheme depicting the UTR-exon-intron structure of *CA1.2* and *CA1_ID11* transcript isoforms. UTRs and exons, lower and higher rectangles, respectively; introns, lines with arrowheads. **(C)** Alignment of the translated proteins encoded by the different isoforms. **(D)** Phenotypic characterization of Col-0 plants grown for 3 weeks under normal, well-watered, conditions, and then subjected to drought stress by withholding water for 20 days. Col-0 and mutant plants were grown in separate pots, but randomized in the same container. **(E)** Characterization of Col-0 and *ca* plants grown under short-day conditions. The water status was controlled by watering pots to the same pot weight (150 g) until the weight was gradually reduced to 110 g. After 44 days, pots were gradually re-watered. The re-watered Col-0 plants died while *ca* plants were still viable as indicated by detectable photosynthetic activity (maximum quantum yield of photosystem II, Fv/Fm). **(F)** Phenotypic characterization and Fv/Fm PAM images of 49-day-old Col-0 and *ca* plants treated as in D.

Second, mRNA levels of *CA1* were markedly decreased at both the transcript isoform and gene levels (**Figure 7A**, **Table 1**). The expression of *CA1_ID12* and *CA1_P4* was found to be very low under all conditions. Among the isoforms, *CA1_ID11* was the most highly expressed under control conditions, but it was rapidly down-regulated under drought stress (**Figure 7A**). The *CA1_ID11* isoform encodes a slightly shorter protein than the *CA1.2* isoform (**Figures 7B, C**). A previous study on the characterization of *CA1* variants identified three of the transcript variants identified in this experiment. The study found that the CA1_ID11 protein was mainly localized in the envelope, while the *CA1.2*-derived protein seemed to be evenly distributed in the chloroplast stroma (Shen et al., 2021), suggesting that particularly the envelope-localized CA1 is of importance for the drought response.

Phosphorylation of CA1 may be important for drought stress adaptation in *Brassica napus* (Wang et al., 2016), and drought experiments with maize *ca1 ca2* plants suggest a role for CAs in water use efficiency (Kolbe et al., 2018). Also, CO_2_-induced stomatal closure is altered in an Arabidopsis *ca1 ca4* double mutant. However, single T-DNA mutants in *CA1*, *CA4*, and *CA6* did not show strong phenotypes in CO_2_ responses (Hu et al., 2010). Based on the strong reduction in *CA1* transcript isoforms that we observed, we wanted to test whether a single *ca1* mutant of Arabidopsis might also exhibit altered tolerance to drought. To this end, Col-0, *ca1*, and, because *CA2* transcript levels were also greatly reduced under drought (**Figure 7A**), *ca2* plants were grown for three weeks under normal growth conditions and then not irrigated for 20 days. Interestingly, all *ca1* and *ca2* plants survived, while all Col-0 plants died (**Figure 7D**). To investigate this further, we conducted a second drought experiment in which we exposed Col-0 and the mutants to the same drought conditions by controlling irrigation through adjustment of pot weights (PSI system described in Materials and Methods; **Figure 7E**). Remarkably, the Col-0 plants died after drought treatment and re-watering, whereas the *ca1* and *ca2* mutants still exhibited high photosynthetic activity, as shown by measuring the maximum quantum yield of photosystem (PS) II (Fv/Fm; **Figures 7E, F**).

In summary, the candidates we identified may provide a solid basis for further exploration of responses to drought stress, and we present a summary of the candidates of interest in **Table 1**. In particular, *FLM* isoform switching offers a possible explanation for the phenotype of early flowering under drought stress. Overall, the results suggest that the identified transcript isoforms may play an important role in the plant response to drought stress, and further studies could provide valuable insights into the mechanisms underlying this response.

## 4 Discussion

As a drought set-up, we used soil-grown plants from which water was withheld for various periods. Many drought models are available, but there is no “ideal” model that can meet all the requirements for drought studies, and individual methods have their particular limitations (Lawlor, 2013). Aqueous or agar media are more stable and reproducible (Ito et al., 2006; Geissler and Wessjohann, 2011; Park et al., 2013) than soil-based models. However, the latter more closely mimic actual drought conditions in the field, which makes it the preferred system in many studies. One typical soil-based model is the “water-withheld-setup”, in which plants are deprived of water until symptoms of wilting are observed (Ali et al., 2020). We deliberately chose this system, because it is accessible to all laboratories. Moreover, field conditions do not allow for the strict control of water availability. To test the robustness of this system, we followed the method used by Kim et al. (2017). Despite the usage of different methodologies to analyze transcriptomes (RNA-Seq vs. microarrays), the transcriptome changes (at the gene level) induced by prolonged drought stress detected in the two studies were similar (see **Figure 1**).

### 4.1 Organellar (post)transcriptomes under stress

Mitochondria and plastids integrate signals to link metabolic processes with environmental sensing (Dopp et al., 2021). Despite the transfer of most of their genes to the nucleus during evolution, these organelles retain their own gene-expression machinery, thus necessitating tight coordination between organellar and nuclear gene expression (OGE and NGE), which is achieved by retrograde and anterograde signaling (Kleine and Leister, 2016; Dopp et al., 2021). Indeed, under drought, adjustment of organellar and nuclear genomes became apparent at the whole-gene expression level. Drought stress has an overall negative impact on chloroplast transcript accumulation (see **Figure 2**), which is in line with the down-regulation of nuclear transcripts coding for proteins involved in chloroplast translation and photosynthesis (see **Supplementary Table 3**). Interestingly, mitochondrial transcripts tended to be slightly upregulated (see **Figure 3**), e.g., those encoding components of succinate-dehydrogenase complex II and enzymes of the tricarboxylic acid cycle (TCA).

Apart from sensing environmental changes, chloroplasts are also targets of adverse conditions (Kleine et al., 2021), and it has been shown that the accumulation of specific plastid RNAs is regulated in mutants with photosynthetic defects and in plants exposed to stresses (see for instance (Cho et al., 2009). Much effort has been put into the investigation of changes in NGE in response to altered organellar states, at both the whole-gene and more recently post-transcriptome levels (Petrillo et al., 2014; Fang et al., 2019; Tiwari et al., 2020). However, only one study of Arabidopsis organellar post-transcriptomes in chloroplasts has been reported previously (Castandet et al., 2016) and there are none for mitochondria. In the context of the development of a chloroplast RNA-Seq bioinformatics pipeline, an analysis of suitable (i.e., RNA-Seq after ribosomal RNA depletion) published transcriptomes (Di et al., 2014) revealed a global reduction in chloroplast splicing and editing efficiency, and an increased abundance of transcripts in response to heat, while short-term “drought” treatment (3 and 12 h in 300 mM mannitol) had negligible effects on the chloroplast (post)transcriptome (Castandet et al., 2016). It should be noted that the sequencing data used by Castandet et al. (2016) were generated in only one copy, and these data may not be statistically sound. Defects in editing or splicing can have profound effects on plant development, and even result in lethality (Kleine and Leister, 2015), and altered adaptability to environmental stresses (Leister et al., 2017; Zhang et al., 2020). Therefore, we wanted to investigate the reverse case: What impact does drought have on the overall accumulation, splicing and editing of organellar transcripts?

Interestingly, levels of mitochondrial and chloroplast RNAs under drought were inversely regulated. While amounts of mitochondrial RNAs tended to rise (see **Figure 3**), drought had a profoundly negative impact on the accumulation of chloroplast transcripts (see **Figure 2**). The most severely affected transcripts – those encoding a component of the large ribosomal subunit (rpl23/uL23c) and the D3, F and K subunits of the NAD(P)H dehydrogenase (NDH) complex – were reduced to 3% of their starting amounts. In contrast, transcripts coding for mitochondrial NADH dehydrogenase subunits increased during prolonged drought stress, and the two most repressed mitochondrial transcripts after 15 days of drought stress were *ATP synthase subunit 1* (*atp1*; 0.3%) and *rpsL2* (17.8%). The function of the NDH complex has been extensively discussed and has yet to be resolved (Labs et al., 2016; Yamori and Shikanai, 2016). In particular, the role of the NDH complex under different stress conditions remains controversial (Yamori and Shikanai, 2016; Lenzen et al., 2020).

Editing capacity in Col-0 mitochondria under drought was not changed (see **Figure 3**), but a reduced editing capacity was observed especially for the chloroplast *ndhD*, *ndhF*, *rpl23*/*uL23c* transcripts, and *rpoA* and *rpoB* transcripts coding for the α and β subunits of the plastid-encoded RNA polymerase (PEP) (see **Figure 2**). Note here that *ndhD*, *rpoA* and *rpoB* are not among the most reduced transcripts, implying that editing of these transcripts plays a more prominent role than their accumulation under drought conditions. Furthermore, editing capacity was maintained or even enhanced until the sudden drop occurred after 15 days of drought stress. This explains the maintenance of relatively high levels of *psbA*, -*C* and -*D* transcripts, because non-functional editing of *rpoB* transcripts leads to a significant decrease in PEP activity (Zhou et al., 2009).

In mitochondria, the only detected down-regulation of splicing efficiency was transiently observed for *cytochrome oxidase 2* (*cox2*), *nad1* and *nad7-2*, and this was slightly enhanced after 15 days (see **Figure 3**). In chloroplasts also, splicing efficiency was not generally reduced, but slightly enhanced after 15 days of drought (see **Figure 2**). Thus, organelle-specific splicing seems to be of marginal importance for the drought stress response.

### 4.2 Alternative splicing enhances proteome diversity to counteract stress responses

Differential alternative splicing (DAS), which enables multiple transcripts (and therefore proteins with different properties) to be produced from single genes, turns out to be an important aspect of responses to adverse conditions (Laloum et al., 2018) – and components of the spliceosome (which mediates DAS) are known to be altered during drought stress (Marondedze et al., 2019). Here, we identified nearly 1,500 DAS genes (see **Figure 4** and **Supplementary Table 8**), of which 42% were regulated solely at the level of alternative splicing (AS) rather than by alteration of transcription rates, so that these transcripts would have gone unnoticed in a microarray-based approach. Some of the identified genes have already been shown to encode proteins involved in stress pathways. Thus, phyB, LESION SIMULATING DISEASE 1 (LSD1) and GLYCINE RICH PROTEIN 7 (GRP7) have previously been implicated in survival of Arabidopsis plants under drought stress (Gonzalez et al., 2012; Szechynska-Hebda et al., 2016) or higher grain yields of rice under drought conditions (Yang et al., 2014). However, it was not known until now that these effects are mediated by different transcript isoforms. Interestingly, GRP7 itself regulates AS (Streitner et al., 2012). Moreover, CONSTITUTIVELY STRESSED 1 (COST1) regulates autophagy to enhance plant drought tolerance (Bao et al., 2020). In this respect it is remarkable that *ATG8B*, -*E* and -*F*, and *ATG13* (all of which code for AUTOPHAGY ubiquitin-like superfamily proteins) are subject to DAS under drought.

One known example of DAS under drought is that of the ZINC-INDUCED FACILITATOR-LIKE 1 (ZIFL1) transporter. The full-length isoform is localized in the tonoplast of root cells and regulates transport of auxin, but a truncated variant is targeted to the plasma membrane of leaf stomatal guard cells and mediates drought tolerance (Remy et al., 2013). A homolog, *ZINC-INDUCED FACILITATOR 2* (*ZIF2*), is known to produce two splice variants, *ZIF2.1* and *ZIF2.2*, which encode the same proteins, but an intron retention event in the 5´UTR in *ZIF2.2* enhances translation in a zinc-responsive manner and promotes zinc tolerance (Remy et al., 2013). This makes *ZIF2* an attractive candidate – among the many promising DAS genes expressed under drought conditions – for further investigation of the contribution of DAS forms to drought tolerance. Another interesting candidate is *ALDEHYDE DEHYDROGENASE 7B4* (*ALDH7B4*; 3000-fold induced after 12 and 15 days) which also undergoes DAS under drought. Indeed, *aldh7b4* knockout mutants exhibit higher sensitivity to dehydration and salt than do wild-type plants (Kotchoni et al., 2006).

Vascular land plants contain α-, β-, and γ-CAs (carbonic anhydrases), and CAs catalyse the interconversion of carbon dioxide and bicarbonate (Hewett-Emmett and Tashian, 1996). *CARBONIC ANHYDRASE1* (*CA1*, *ßCA1*) mRNA levels were reduced to 0.005% of control levels after 15 days of drought stress (see **Supplementary Table 3**), and the *ca1* mutant is more drought tolerant (see **Figure 7**). In particular, one isoform, *CA1_ID11*, was rapidly down-regulated under drought stress, suggesting that the CA1 protein produced by *CA1_ID11* may be important for drought response. The *CA1_ID11* isoform encodes a slightly shorter protein than the *CA1.2* isoform (**Figure 7C**), and the protein produced by *CA1_ID11* was mainly localized in the envelope, whereas the protein derived from *CA1.2* appeared to be evenly distributed in the chloroplast stroma (Shen et al., 2021). The importance of CA1 localization was demonstrated by Hines et al. (2021): Tobacco stromal CA1 (and CA5) isoforms play no role in photosynthesis but do play a role in plant development, whereas no such function could be ascribed to cytosolic CA1. Arabidopsis contains at least two stromal ßCAs, ßCA1 and ßCA5, while ßCA2 is one of the most abundant isoforms in the cytosol and, together with ßCA4, is required for optimal growth under low CO_2_ (DiMario et al., 2016). Remarkably, CA1 has been found to translocate from tobacco chloroplasts to the cytosol under drought stress (Li et al., 2020), and we observed that plants lacking ßCA2 are as drought tolerant as the *ca1* mutant. By studying a *ca1 ca2* mutant of maize, it was suggested that CA1 and CA2 also play a role in water use efficiency in a C4 plant, which is likely mediated by an altered stomatal response (Kolbe et al., 2018). It will be interesting to investigate in the future which CA isoforms at which sites determine drought tolerance and whether this mechanism is conserved in C3 and C4 plants.

### 4.3 Early flowering under drought stress is most probably caused by a lack of functional *FLM* and/or reduced *SVP* transcripts

The drought escape (DE) strategy involves an earlier switch from vegetative to reproductive development, enabling reproduction before severe water deficit prohibits plant survival (Kenney et al., 2014). Early flowering as a DE mechanism is extremely important and research on the topic has a very long history (Shavrukov et al., 2017). Under a 12 h/12 h light/dark regime, ecotypes with low expression of *FRIGIDA* (*FRI*), or a null *FRI* allele as in Col-0, confer early flowering under drought (Lovell et al., 2013). Also, in our experimental setup in which we used long-day conditions, Col-0 plants began to flower earlier under drought. In Arabidopsis, flowering is ultimately achieved by activating expression of the gene *FLOWERING LOCUS T* (*FT*) (Searle and Coupland, 2004; **Figure 6**). Accordingly, in our experimental setup, *FT* mRNA levels are strongly elevated under drought treatment (40-fold induction after 12 and 15 days, see **Figure 5**, **Supplementary Table 3**). The DE response is dependent on the photoperiod – at least for the Arabidopsis Col-0 and L*er* ecotypes – because short-day-grown plants do not flower earlier under drought stress (Riboni et al., 2013). In addition to FT, the photoperiodic pathway is characterized by two other key components, GIGANTEA (GI) and CONSTANS (CO) (Putterill et al., 1995; Fowler et al., 1999). Complete absence of the DE response was observed in *gi* mutants in both the Col-0 and L*er* backgrounds, but this response does not appear to require the activity of CO (Riboni et al., 2013), which is a transcriptional regulator of *FT* that acts downstream of GI. Correspondingly, *GI* mRNA levels are 5-fold induced and those of *CO* barely detectable under our drought conditions (**Figure 6**). GI also acts in CO-independent branches by either directly binding to the FT promoter and competing with some repressors of *FT* such as SHORT VEGETATIVE PHASE (SVP), or promoting *miR172* expression which inhibits the expression of AP2-like transcription factors, such as *TOE1*, *TPL*, *SMZ*, thus repressing *FT* transcription [summarized in Kim (2020)]. Under our drought conditions, *TOE1* and *SMZ* mRNAs are barely detectable, while levels of *TPL* are elevated 11-fold (**Figure 6**). Amounts of *SVP* mRNAs are reduced to 20% of their starting levels in accordance with the elevated *GI* and *FT* levels and this mechanism might contribute to the earlier flowering phenotype under long-day drought conditions. However, the alternative splicing and isoform switching (IS) of *FLOWERING LOCUS M* (*FLM*) (see **Figure 5**), another repressor of *FT* transcription which also acts independently of CO (Balasubramanian et al., 2006), is particularly noteworthy. FLM and SVP also participate in the autonomous (Scortecci et al., 2001) and the thermosensory (Balasubramanian et al., 2006) flowering pathways. *FLM* undergoes temperature-dependent alternative splicing, and the roles of the different isoforms have been extensively and controversially discussed in the literature. It was proposed that the major isoforms, *FLM-β* and *FLM-δ*, which result from the alternative usage of exons 2 (*FLM-β*) and 3 (*FLM-δ*) (Lee et al., 2013; Pose et al., 2013) might compete for interaction with SVP, and the SVP-FLM-β complex is predominantly formed at low temperatures and prevents precocious flowering (Pose et al., 2013). One model for flowering at high temperatures suggests that degradation of SVP reduces the abundance of the SVP-FLM-β repressor complex (Lee et al., 2013), the other model is based on the idea that a higher *FLM-δ*/*FLM-β* ratio favors the formation of an SVP–FLM-δ complex that is impaired in DNA binding and acts as a dominant-negative activator of flowering at higher temperatures (Pose et al., 2013). We assume that under (our) drought conditions a higher FLM-*β*/FLM-*δ* ratio is unlikely to be the trigger for the flowering DE response. Our qRT-PCR data suggest that amounts of the *FLM-β* isoform increase after 9 days of drought stress, while RNA-Seq analysis indicates that levels of FLM-ß are slightly reduced relative to the onset of drought treatment (see **Figure 5**). This discrepancy can be explained by accumulation of other non-functional isoforms containing exon 2 or exon 3, respectively (see also Sureshkumar et al., 2016), which are amplified by qRT-PCR owing to the frequent use of primer designs in which the reverse primer is situated in exon 2 (*FLM-ß*) or 3 (*FLM-δ*), respectively (for example also used in Pose et al., 2013). Although the *FLM-β*/*FLM-δ* ratio rises especially after 12 days of drought stress, the accumulation of these forms is very low in comparison to that prior to drought stress, and both are nearly undetectable anymore after 15 days. Moreover, the *FLM_P10* form, which is predominantly produced under prolonged drought stress, contains a premature stop codon that would encode a protein of only 62 amino acids (see **Supplementary Table 9**). Indeed, under high temperatures too, *FLM* expression is downregulated by AS coupled with nonsense-mediated mRNA decay (AS-NMD), and the majority of non-canonical *FLM* transcripts contained premature termination codons that would result in truncated proteins of less than 100 amino acids (Sureshkumar et al., 2016). Moreover, allelic variation at *FLM* modulates plant growth strategy observable across thousands of plant species. The authors found that functional differences at *FLM* rely on a single intronic substitution, disturbing transcript splicing and leading also to the accumulation of non-functional *FLM* transcripts (Hanemian et al., 2020). All in all, we suggest that drought-mediated early flowering under long-day conditions is caused by the absence of functional FLM protein and not by the ratio of isoforms.

The decision whether to investigate under long- or short-day conditions is a very important one, since it may result in contrasting outcomes. In relation to drought research, SVP was shown to be up-regulated at the mRNA level and to confer drought tolerance in short-day-grown plants (Bechtold et al., 2016; Wang Z. et al., 2018). However, in our long-day kinetic experiment, SVP is downregulated at all times, and would not have been discovered as a positive factor in drought stress.

Moreover, our transcriptomic data sets provide a rich resource (see also **Table 1**) for the elucidation of the many facets of drought stress mechanisms. In particular, evaluation of the relative contributions of different splicing isoforms to drought tolerance will increase our understanding of the modulation of abiotic stress responses, thus enabling the development of new strategies to improve plant performance under adverse environmental conditions.

## Data availability statement

The datasets presented in this study can be found in online repositories. The names of the repository/repositories and accession number(s) can be found in the article/**Supplementary Material**.

## Author contributions

Conceptualization, TK and DX; Methodology, DX, QT, PX, and TK; Validation, DX and QT; Formal Analysis, TK; Investigation, DX, QT, and TK; Resources, DL, AS and TK; Writing – Original Draft, TK; Writing – Review & Editing, QT, DX, DL, AS and TK; Funding Acquisition, DL and TK Supervision, TK. All authors contributed to the article and approved the submitted version.
