# Supplementary material for: Response of the organellar and nuclear (post)transcriptomes of Arabidopsis to drought
Source: Front Plant Sci. 2023 Jul 17;14:1220928. doi: 10.3389/fpls.2023.1220928 (PMC10387551; doi:10.3389/fpls.2023.1220928)
Supplement: Supplementary file 1 [file DataSheet_1.pdf]

Supplementary Material

Response of the organellar and nuclear (post)transcriptomes of Arabidopsis to drought stress

Duorong Xu[1], Qian Tang[1], Ping Xu[2], Anton R Schäffner[2], Dario Leister[1], and Tatjana Kleine[1,*]

*** Correspondence:**
Dr. Tatjana Kleine

Ludwig-Maximilians-Universität München (LMU)
Department Biologie I, Botanik; Großhaderner Str. 2, D-82152 Planegg-Martinsried
Phone: +49-89/2180 74554; Fax: +49-89/2180 74599; E-mail: tatjana.kleine@lmu.de

1 Supplementary Figures and Tables

1.1 Supplementary Figures

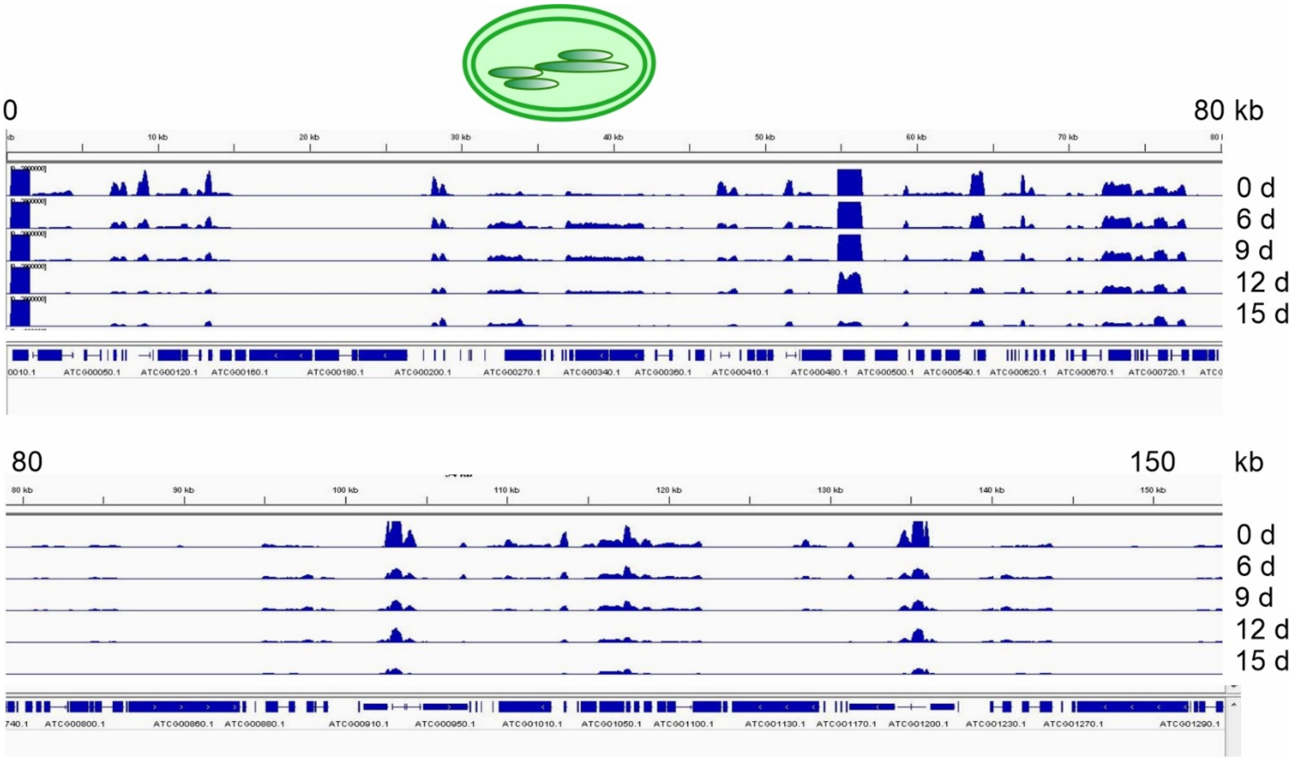

Supplementary Figure 1. Distribution of lncRNA-Seq reads across the chloroplast genome. The normalized read depths of transcripts detected in Col-0 plants under control (0 d) and drought conditions were visualized with the Integrative Genomics Viewer (IGV).

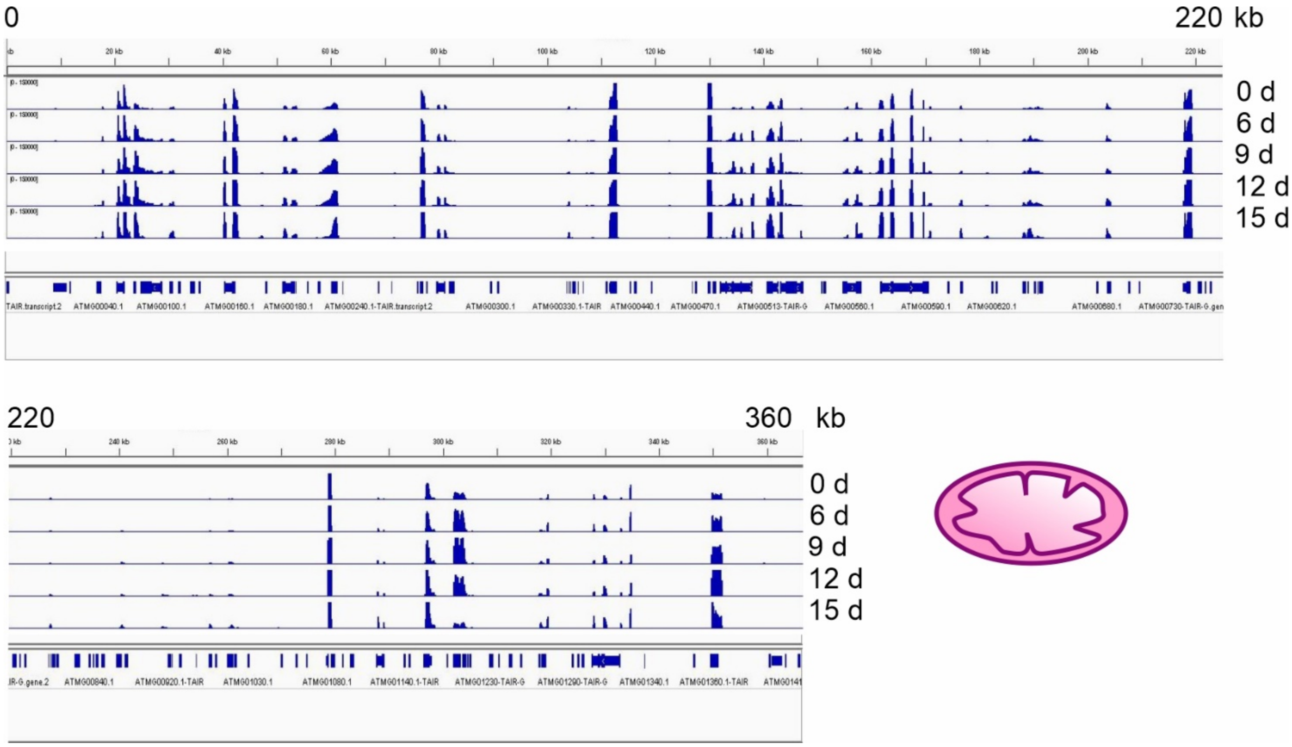

Supplementary Figure 2. Distribution of lncRNA-Seq reads across the mitochondrial genome. The normalized read depths of transcripts detected in Col-0 plants under control (0 d) and drought conditions were visualized with the Integrative Genomics Viewer (IGV).

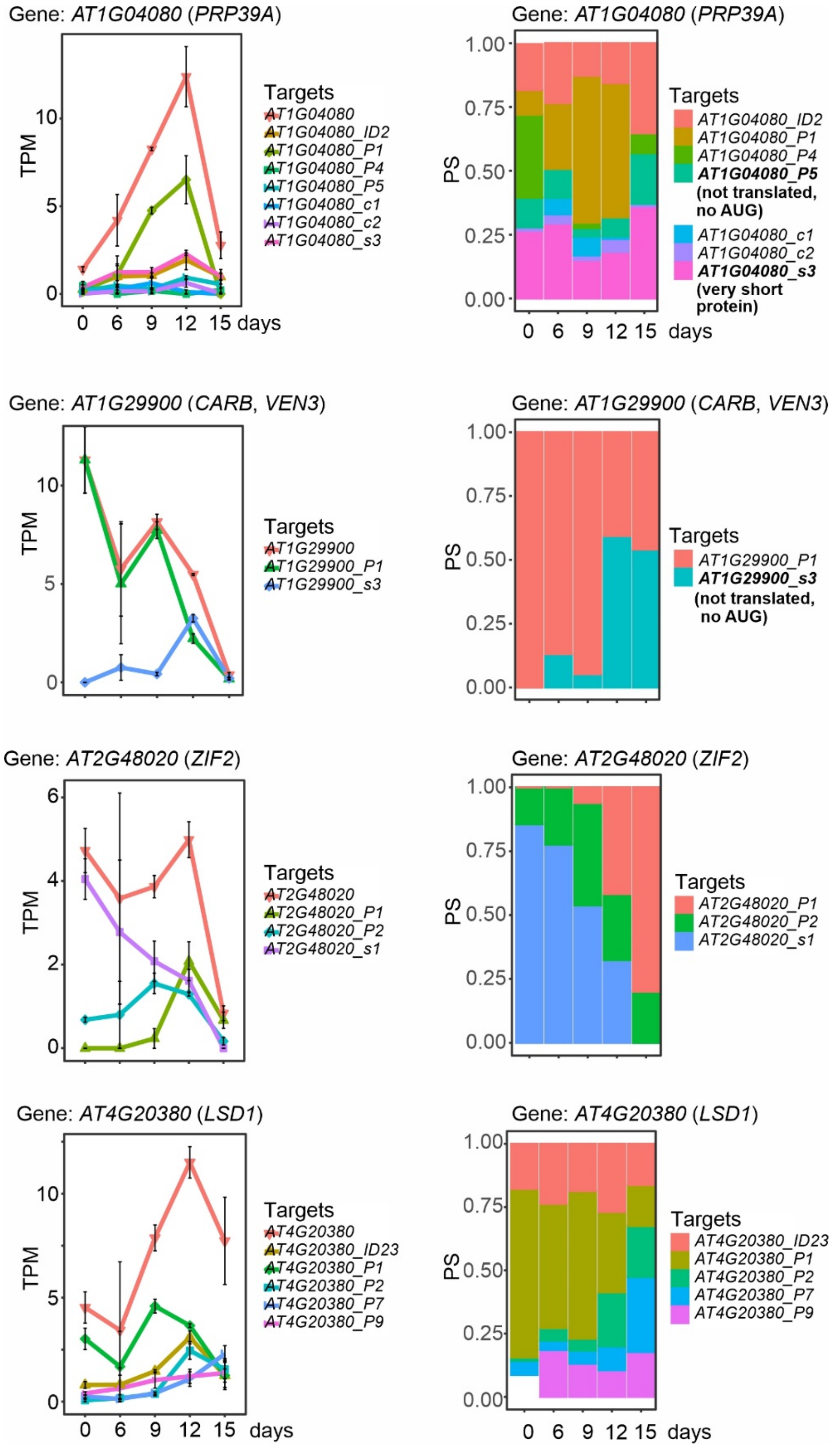

Supplementary Figure 3. Illustrations of further isoform switching (IS) events. Expression profiles of *PRP39A*, *CARB*, *ZIG2* and *LSD1* at the whole-gene level and at the level of detected transcript isoforms are shown. PS, percentage of expressed transcripts spliced; TPM, transcripts per million reads.

1.2 Supplementary Tables

Supplementary Table 1. Primers used in this study.

Atg number	Description	Primer sequence (from 5' to 3')
**qRT-PCR**		
AT1G77080	*FLM-RT_F*	TCGCTGTTGTCGTCGTATCTGC
AT1G77080	*FLM-e1-e3-RT_R*	CGGCTTGAACAGCGCTTCTATCTC
AT1G77080	*FLM-e1-e2-RT_R*	CAATGATCTTGGAAATGTCGTCACCG
AT1G77080	*FLM-intr1-RT_R*	GAAGCTTCTATATGGAGAAAGTAA

In separate files:

Supplementary Table 2. Sequencing depth of lncRNA-Seq experiments. Long non-coding RNA sequencing (lncRNA-Seq) was performed with RNA isolated from 3-week-old Col-0 (Col) plants grown under optimal conditions (time-point 0 days; 0d) and after water had been withheld for 6, 9, 12 and 15 days, respectively (6d, 9d, 12d and 15d). Sequences were mapped and analyzed as described in Materials and Methods.

Supplementary Table 3. Genes differentially expressed under drought stress. Long non-coding RNA sequencing (lncRNA-Seq) was performed with RNA isolated from 3-week-old Col-0 (Col) plants grown under optimal conditions (time-point 0 days; 0d) and after water had been withheld for 6, 9, 12 and 15 days, respectively (6d, 9d, 12d and 15d). Sequences were mapped and analyzed with the 3D RNA-Seq pipeline as described in Materials and Methods.

Supplementary Table 4. Drought-stress marker genes. Lists of genes whose transcripts are regulated in the same direction (up or down) after 9, 12 and 15 days of drought stress in our dataset as well as in Kim et al. (2017).

Supplementary Table 5. Gene Ontology (GO) analysis of the nine clusters listed in Figure 1. Biological process (BP) and cellular component (CC) categories were identified with DAVID (Huang da *et al.*, 2009) and REVIGO (Supek *et al.*, 2011).

Supplementary Table 6. Abundance of chloroplast and mitochondrial transcripts. **(A)** $\log_2$ fold changes of RNAs encoded by the chloroplast genome. Note that analysis of tRNAs was excluded. **(B)** $\log_2$ fold changes of RNAs encoded by the mitochondrial genome.

Supplementary Table 7. Differentially expressed transcript isoforms under drought stress.

Supplementary Table 8. List of differentially alternatively spliced (DAS) genes under drought stress.

Supplementary Table 9. List of genes that showed isoform switches (ISs) during drought stress.
